# (*E*)-1-Ethyl-4-oxo-*N*′-(4-pyridylmethyl­ene)-1,4-dihydroquinoline-3-carbo­hydrazide

**DOI:** 10.1107/S160053680903654X

**Published:** 2009-09-16

**Authors:** Fernanda da C. Santos, Pedro Netto Batalha, Anna Claudia Cunha, Rafael A. Alão, Vitor Francisco Ferreira, Maria Cecilia B. V. de Souza, Sauli Santos

**Affiliations:** a Instituto de Química, Universidade Federal Fluminense, Nitéroi - Rio de Janeiro, Brazil; b Coordenação de Física, Campus de Jataí, Universidade Federal de Goiás, Jataí - Goiás, Brazil

## Abstract

In the title compound, C_18_H_16_N_4_O_2_, the plane defined by the ethyl C atoms and the attached N atom is inclined to the adjacent pyridine ring at an angle of 67.87 (16)°. The dihedral angle between the two heterocyclic rings is 3.33 (16)°. The mol­ecular conformation is stabilized by an intra­molecular N—H⋯O hydrogen bond and the crystal structure by inter­molecular C—H⋯O hydrogen bonds, forming a one-dimensional structure.

## Related literature

For the biological properties of oxoquinoline derivatives, see: Van Bambeke *et al.* (2005 ▶); Canuto *et al.* (2007 ▶); Lucero *et al.* (2006 ▶). For their potential use in the treatment of fungal and viral infections, see: Brideau *et al.* (2002 ▶); Souza *et al.* (2008 ▶) and in cancer chemotherapy, see: Chu *et al.* (1992 ▶). For acyl­hydrazones and their anti­leishmanial activity, see: Bernadino *et al.* (2006 ▶); Cunha *et al.* (2003 ▶). For bond-length data, see: Allen *et al.* (1987 ▶).
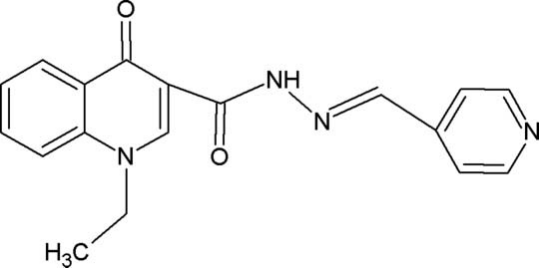


## Experimental

### 

#### Crystal data

C_18_H_16_N_4_O_2_
*M* *_r_* = 320.35
Monoclinic, 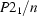

*a* = 7.6460 (12) Å
*b* = 19.205 (2) Å
*c* = 10.7050 (9) Å
β = 99.722 (10)°
*V* = 1549.4 (3) Å^3^
*Z* = 4
Mo *K*α radiation
μ = 0.09 mm^−1^
*T* = 293 K
0.5 × 0.2 × 0.2 mm

#### Data collection

Nonius KappaCCD diffractometer
Absorption correction: none
7914 measured reflections
2586 independent reflections
2001 reflections with *I* > 2σ(*I*)
*R*_int_ = 0.019

#### Refinement

*R*[*F* ^2^ > 2σ(*F* ^2^)] = 0.041
*wR*(*F* ^2^) = 0.102
*S* = 1.07
2586 reflections
222 parameters
H atoms treated by a mixture of independent and constrained refinement
Δρ_max_ = 0.13 e Å^−3^
Δρ_min_ = −0.15 e Å^−3^

### 

Data collection: *COLLECT* (Nonius, 2000 ▶); cell refinement: *HKL* *SCALEPACK* (Otwinowski & Minor 1997 ▶); data reduction: *HKL* *DENZO* (Otwinowski & Minor 1997 ▶) and *SCALEPACK*; program(s) used to solve structure: *SIR2002* (Burla *et al.*, 2003 ▶); program(s) used to refine structure: *SHELXL97* (Sheldrick, 2008 ▶); molecular graphics: *ORTEP-3* (Farrugia, 1997 ▶) and *Mercury* (Macrae *et al.*, 2008 ▶); software used to prepare material for publication: *WinGX* publication routines (Farrugia, 1999 ▶).

## Supplementary Material

Crystal structure: contains datablocks I, global. DOI: 10.1107/S160053680903654X/wn2340sup1.cif

Structure factors: contains datablocks I. DOI: 10.1107/S160053680903654X/wn2340Isup2.hkl

Additional supplementary materials:  crystallographic information; 3D view; checkCIF report

## Figures and Tables

**Table 1:** Hydrogen-bond geometry (Å, °)

*D*—H⋯*A*	*D*—H	H⋯*A*	*D*⋯*A*	*D*—H⋯*A*
N3—H3*N*⋯O1	0.92 (2)	1.85 (2)	2.639 (2)	143.1 (17)
C10—H10*A*⋯O2^i^	0.97	2.46	3.398 (2)	163

Symmetry code: (i) 
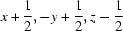
.

**Fig. 1.**
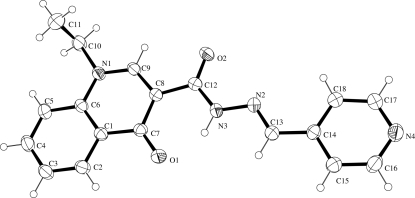
The molecular structure of the title compound showing the atom labelling scheme. Displacement ellipsoids are drawn at the 50% probability level. Hydrogen atoms are shown as spheres of arbitrary radius.

**Fig. 2.**
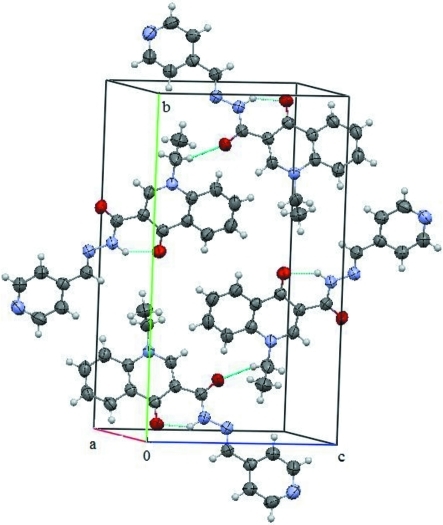
The molecular packing, with hydrogen bonds drawn as dashed lines.

## supplementary crystallographic information

### Comment

Since the discovery of nalidixic acid, the parent compound of the 1,4-dihydro-4-oxoquinoline antibiotics, the molecular structures of oxoquinolines have been extensively modified to improve their pharmacological properties and pharmacokinetic profiles (Van Bambeke et al., 2005). 1,4-Dihydro-4-oxoquinolines have a broad antimicrobial spectrum, are orally and parenterally active and, apart from a few exceptions, are non-toxic compounds. Therefore, they are important agents against microbial pathogens. They have also been considered for the treatment of fungal and viral (Brideau et al., 2002; Souza et al., 2008) infections and for cancer chemotherapy (Chu et al., 1992). Our research group has designed and synthesized new oxoquinoline derivatives, such as ribonucleosides (Canuto et al., 2007) and acyclonucleosides (Lucero et al., 2006). These compounds exhibited interesting activity against HIV-1 and HSV viruses, respectively.

Continuing our interest in the chemistry of oxoquinolinic molecules, we decided to prepare a congener series of new acylhydrazones (Cunha et al., 2003) and test them for antileishmanial activity. These studies showed that the hydrazone group plays an important role in antileishmanial activity (Bernadino et al., 2006). Among these compounds, (E)-1-ethyl-N'-[(pyrid-4'-yl)methylene]-1,4-dihydro-4- oxoquinoline-3-carbohydrazide, was found to exhibit a significant activity against Leishmania amazonensis.

As an extension of our work on the structural characterization of oxoquinolinic derivatives (Canuto et al., 2007; Lucero et al. 2006). the crystal structure of the title compound is reported here. In the title compound (Fig. 1), the dihedral angle between the C1,C6-C9/N1 ring and the C14-C18/N4 pyridine ring is 3.33 (16)°. The plane defined by N1,C10,C11 is inclined to the C1,C6-C9/N1 ring at an angle of 67.87 (16)°. The torsion angles C8—C12—N3—N2, C12—N3—N2—C13 and N3—N2—C13—C14 are 0.79 (14), 1.38 (16) and 1.53 (14)°, respectively. All the bond lengths are within normal ranges (Allen et al., 1987). The molecular structure is stabilized by an intramolecular N—H···O hydrogen bond (Table 1). The crystal structure is stabilized by intermolecular C—H···O hydrogen bonds, forming a one-dimensional structure (Table 1 and Fig. 2).

### Experimental

A solution of 1-ethyl-1,4-dihydro-4-oxoquinoline ethyl carboxylate (3.70 mmol) and 3.7 ml of 80% hydrazine monohydrate in 10 ml of dimethylformamide was stirred under reflux for two hours. The reaction mixture was poured into ice, giving rise to a white solid that was collected by filtration, washed with cold ethyl acetate and dried under vacuum, leading to the pure desired 1-ethyl-1,4-dihydro-4-oxoquinoline-3-carbohydrazide, in 90% yield. This carbohydrazide (1.00 mmol) and pyridine-4-carbaldehyde (1.10 mmol) in 5 ml of dimethylformamide and a catalytic amount of 35% HCl were stirred at room temperature. The reaction mixture was poured into ice, leading to a white solid that was collected by filtration, giving (*E*)-1-ethyl-*N*'-[(pyrid-4'-yl)methylene]-1,4-dihydro-4-oxoquinoline-3-carbohydrazide in 83% yield after purification.

### Refinement

The N-bound H atom was located in a difference map and refined freely [to N—H = 0.92 (2) Å]. The other H atoms were positioned with idealized geometry and refined using a riding model, with C—H = 0.93 Å for aryl, 0.97 Å for methylene, and 0.96 Å for methyl H atoms. The constraints *U*_iso_(H) = 1.2*U*_eq_(C) and 1.5*U*_eq_(C) (methyl C) were applied.

### Crystal data

**Table:**

C_18_H_16_N_4_O_2_	*F*(000) = 672
*M**_r_* = 320.35	*D*_x_ = 1.373 Mg m^−^^3^
Monoclinic, *P*2_1_/*n*	Mo *K*α radiation, λ = 0.71073 Å
Hall symbol: -P 2yn	Cell parameters from 7914 reflections
*a* = 7.6460 (12) Å	θ = 5.2–25°
*b* = 19.205 (2) Å	µ = 0.09 mm^−^^1^
*c* = 10.7050 (9) Å	*T* = 293 K
β = 99.722 (10)°	Prism, yellow
*V* = 1549.4 (3) Å^3^	0.5 × 0.2 × 0.2 mm
*Z* = 4	

### Data collection

**Table:**

Nonius KappaCCD diffractometer	2001 reflections with *I* > 2σ(*I*)
Radiation source: fine-focus sealed tube	*R*_int_ = 0.019
graphite	θ_max_ = 25.0°, θ_min_ = 5.2°
Detector resolution: 9 pixels mm^-1^	*h* = −9→9
φ scans and ω scans winth κ offsets	*k* = −22→20
7914 measured reflections	*l* = −12→8
2586 independent reflections	

### Refinement

**Table:**

Refinement on *F*^2^	Primary atom site location: structure-invariant direct methods
Least-squares matrix: full	Secondary atom site location: difference Fourier map
*R*[*F*^2^ > 2σ(*F*^2^)] = 0.041	Hydrogen site location: inferred from neighbouring sites
*wR*(*F*^2^) = 0.102	H atoms treated by a mixture of independent and constrained refinement
*S* = 1.07	*w* = 1/[σ^2^(*F*_o_^2^) + (0.0352*P*)^2^ + 0.5415*P*] where *P* = (*F*_o_^2^ + 2*F*_c_^2^)/3
2586 reflections	(Δ/σ)_max_ < 0.001
222 parameters	Δρ_max_ = 0.13 e Å^−^^3^
0 restraints	Δρ_min_ = −0.14 e Å^−^^3^

### Special details

**Table:**

Geometry. All e.s.d.'s (except the e.s.d. in the dihedral angle between two l.s. planes) are estimated using the full covariance matrix. The cell e.s.d.'s are taken into account individually in the estimation of e.s.d.'s in distances, angles and torsion angles; correlations between e.s.d.'s in cell parameters are only used when they are defined by crystal symmetry. An approximate (isotropic) treatment of cell e.s.d.'s is used for estimating e.s.d.'s involving l.s. planes.

### Fractional atomic coordinates and isotropic or equivalent isotropic displacement parameters (Å^2^)

**Table:**

	*x*	*y*	*z*	*U*_iso_*/*U*_eq_	
C1	0.9168 (2)	0.11495 (9)	0.16391 (15)	0.0361 (4)	
C2	0.8969 (2)	0.07582 (10)	0.05163 (16)	0.0455 (4)	
H2	0.8539	0.0305	0.0514	0.055*	
C3	0.9396 (3)	0.10304 (10)	−0.05772 (17)	0.0516 (5)	
H3	0.9240	0.0768	−0.1318	0.062*	
C4	1.0065 (3)	0.17027 (11)	−0.05650 (17)	0.0521 (5)	
H4	1.0374	0.1886	−0.1302	0.063*	
C5	1.0280 (2)	0.21026 (10)	0.05098 (16)	0.0471 (4)	
H5	1.0740	0.2550	0.0500	0.056*	
C6	0.9804 (2)	0.18344 (9)	0.16269 (15)	0.0374 (4)	
C7	0.8708 (2)	0.08354 (9)	0.27920 (15)	0.0371 (4)	
C8	0.8931 (2)	0.12883 (8)	0.38879 (15)	0.0365 (4)	
C9	0.9529 (2)	0.19544 (9)	0.37882 (15)	0.0401 (4)	
H9	0.9638	0.2235	0.4505	0.048*	
C10	1.0520 (3)	0.29759 (10)	0.27524 (18)	0.0538 (5)	
H10A	1.1500	0.3026	0.2291	0.065*	
H10B	1.0931	0.3118	0.3622	0.065*	
C11	0.9018 (3)	0.34469 (11)	0.2170 (2)	0.0750 (7)	
H11A	0.9448	0.3915	0.2143	0.113*	
H11B	0.8090	0.3432	0.2671	0.113*	
H11C	0.8563	0.3293	0.1324	0.113*	
C12	0.8472 (2)	0.10900 (9)	0.51400 (16)	0.0416 (4)	
C13	0.6773 (2)	−0.04576 (9)	0.61567 (16)	0.0429 (4)	
H13	0.6722	−0.0691	0.5391	0.051*	
C14	0.6223 (2)	−0.08221 (9)	0.72279 (15)	0.0388 (4)	
C15	0.5717 (2)	−0.15131 (9)	0.71190 (17)	0.0494 (5)	
H15	0.5690	−0.1747	0.6356	0.059*	
C16	0.5249 (3)	−0.18542 (10)	0.81527 (19)	0.0544 (5)	
H16	0.4916	−0.2319	0.8057	0.065*	
C17	0.5729 (3)	−0.08884 (11)	0.93622 (18)	0.0551 (5)	
H17	0.5739	−0.0667	1.0136	0.066*	
C18	0.6209 (2)	−0.05045 (10)	0.83932 (17)	0.0479 (4)	
H18	0.6521	−0.0038	0.8514	0.057*	
N1	0.99728 (18)	0.22334 (7)	0.27329 (12)	0.0402 (4)	
N2	0.73182 (18)	0.01676 (7)	0.62429 (13)	0.0419 (4)	
N3	0.7828 (2)	0.04327 (8)	0.51697 (14)	0.0445 (4)	
N4	0.5248 (2)	−0.15582 (9)	0.92715 (15)	0.0541 (4)	
O1	0.81667 (17)	0.02200 (6)	0.27864 (11)	0.0522 (3)	
O2	0.8639 (2)	0.14902 (7)	0.60464 (12)	0.0636 (4)	
H3N	0.776 (2)	0.0178 (10)	0.4436 (19)	0.057 (6)*	

### Atomic displacement parameters (Å^2^)

**Table:**

	*U*^11^	*U*^22^	*U*^33^	*U*^12^	*U*^13^	*U*^23^
C1	0.0335 (8)	0.0403 (10)	0.0342 (9)	0.0054 (7)	0.0048 (7)	−0.0024 (7)
C2	0.0512 (10)	0.0443 (10)	0.0408 (10)	0.0051 (8)	0.0074 (8)	−0.0066 (8)
C3	0.0632 (12)	0.0569 (12)	0.0346 (10)	0.0102 (10)	0.0078 (8)	−0.0070 (8)
C4	0.0603 (12)	0.0623 (13)	0.0366 (10)	0.0074 (10)	0.0163 (8)	0.0047 (9)
C5	0.0523 (11)	0.0506 (11)	0.0409 (10)	0.0003 (9)	0.0151 (8)	0.0035 (8)
C6	0.0360 (9)	0.0428 (10)	0.0343 (9)	0.0027 (7)	0.0088 (7)	−0.0018 (7)
C7	0.0364 (9)	0.0365 (9)	0.0388 (9)	0.0030 (7)	0.0075 (7)	−0.0011 (7)
C8	0.0391 (9)	0.0362 (9)	0.0354 (9)	0.0013 (7)	0.0101 (7)	−0.0018 (7)
C9	0.0456 (10)	0.0427 (10)	0.0342 (9)	−0.0017 (8)	0.0132 (7)	−0.0055 (7)
C10	0.0727 (13)	0.0478 (11)	0.0452 (10)	−0.0231 (10)	0.0227 (9)	−0.0063 (9)
C11	0.114 (2)	0.0441 (12)	0.0719 (15)	−0.0014 (13)	0.0312 (14)	0.0043 (11)
C12	0.0491 (10)	0.0379 (10)	0.0403 (10)	0.0021 (8)	0.0144 (8)	−0.0024 (8)
C13	0.0520 (10)	0.0398 (10)	0.0368 (9)	−0.0028 (8)	0.0071 (8)	−0.0011 (7)
C14	0.0380 (9)	0.0378 (9)	0.0394 (9)	0.0020 (7)	0.0029 (7)	0.0047 (7)
C15	0.0611 (12)	0.0424 (10)	0.0440 (10)	−0.0037 (9)	0.0066 (9)	0.0000 (8)
C16	0.0643 (13)	0.0409 (11)	0.0573 (12)	−0.0057 (9)	0.0084 (10)	0.0082 (9)
C17	0.0720 (13)	0.0519 (12)	0.0446 (11)	0.0025 (10)	0.0191 (9)	0.0019 (9)
C18	0.0584 (11)	0.0402 (10)	0.0457 (10)	−0.0006 (8)	0.0110 (8)	−0.0003 (8)
N1	0.0474 (8)	0.0392 (8)	0.0361 (8)	−0.0068 (6)	0.0128 (6)	−0.0033 (6)
N2	0.0500 (9)	0.0395 (9)	0.0381 (8)	−0.0008 (7)	0.0125 (6)	0.0029 (6)
N3	0.0613 (10)	0.0397 (9)	0.0353 (8)	−0.0056 (7)	0.0158 (7)	0.0001 (7)
N4	0.0598 (10)	0.0525 (10)	0.0521 (10)	0.0032 (8)	0.0149 (8)	0.0113 (8)
O1	0.0732 (9)	0.0387 (7)	0.0464 (7)	−0.0095 (6)	0.0147 (6)	−0.0056 (5)
O2	0.1082 (11)	0.0441 (8)	0.0471 (8)	−0.0128 (7)	0.0379 (7)	−0.0102 (6)

### Geometric parameters (Å, °)

**Table:**

C1—C6	1.403 (2)	C10—H10B	0.9700
C1—C2	1.404 (2)	C11—H11A	0.9600
C1—C7	1.469 (2)	C11—H11B	0.9600
C2—C3	1.371 (2)	C11—H11C	0.9600
C2—H2	0.9300	C12—O2	1.227 (2)
C3—C4	1.388 (3)	C12—N3	1.357 (2)
C3—H3	0.9300	C13—N2	1.269 (2)
C4—C5	1.370 (2)	C13—C14	1.465 (2)
C4—H4	0.9300	C13—H13	0.9300
C5—C6	1.405 (2)	C14—C15	1.382 (2)
C5—H5	0.9300	C14—C18	1.390 (2)
C6—N1	1.398 (2)	C15—C16	1.384 (3)
C7—O1	1.2518 (19)	C15—H15	0.9300
C7—C8	1.447 (2)	C16—N4	1.326 (2)
C8—C9	1.369 (2)	C16—H16	0.9300
C8—C12	1.491 (2)	C17—N4	1.337 (2)
C9—N1	1.344 (2)	C17—C18	1.372 (3)
C9—H9	0.9300	C17—H17	0.9300
C10—N1	1.485 (2)	C18—H18	0.9300
C10—C11	1.511 (3)	N2—N3	1.3719 (19)
C10—H10A	0.9700	N3—H3N	0.92 (2)
			
C6—C1—C2	118.79 (15)	C10—C11—H11B	109.5
C6—C1—C7	121.70 (14)	H11A—C11—H11B	109.5
C2—C1—C7	119.52 (15)	C10—C11—H11C	109.5
C3—C2—C1	121.32 (17)	H11A—C11—H11C	109.5
C3—C2—H2	119.3	H11B—C11—H11C	109.5
C1—C2—H2	119.3	O2—C12—N3	123.61 (16)
C2—C3—C4	119.16 (17)	O2—C12—C8	122.81 (15)
C2—C3—H3	120.4	N3—C12—C8	113.58 (14)
C4—C3—H3	120.4	N2—C13—C14	121.97 (15)
C5—C4—C3	121.40 (17)	N2—C13—H13	119.0
C5—C4—H4	119.3	C14—C13—H13	119.0
C3—C4—H4	119.3	C15—C14—C18	116.92 (16)
C4—C5—C6	119.85 (17)	C15—C14—C13	120.43 (16)
C4—C5—H5	120.1	C18—C14—C13	122.65 (16)
C6—C5—H5	120.1	C14—C15—C16	119.58 (17)
N1—C6—C1	119.25 (14)	C14—C15—H15	120.2
N1—C6—C5	121.31 (16)	C16—C15—H15	120.2
C1—C6—C5	119.44 (15)	N4—C16—C15	123.99 (18)
O1—C7—C8	124.40 (15)	N4—C16—H16	118.0
O1—C7—C1	120.64 (14)	C15—C16—H16	118.0
C8—C7—C1	114.96 (14)	N4—C17—C18	124.58 (18)
C9—C8—C7	119.59 (14)	N4—C17—H17	117.7
C9—C8—C12	116.19 (14)	C18—C17—H17	117.7
C7—C8—C12	124.17 (15)	C17—C18—C14	119.08 (18)
N1—C9—C8	124.96 (15)	C17—C18—H18	120.5
N1—C9—H9	117.5	C14—C18—H18	120.5
C8—C9—H9	117.5	C9—N1—C6	119.53 (14)
N1—C10—C11	112.13 (16)	C9—N1—C10	118.75 (14)
N1—C10—H10A	109.2	C6—N1—C10	121.59 (13)
C11—C10—H10A	109.2	C13—N2—N3	115.25 (14)
N1—C10—H10B	109.2	C12—N3—N2	121.35 (15)
C11—C10—H10B	109.2	C12—N3—H3N	116.4 (12)
H10A—C10—H10B	107.9	N2—N3—H3N	122.2 (12)
C10—C11—H11A	109.5	C16—N4—C17	115.84 (16)
			
C6—C1—C2—C3	0.6 (2)	C6—C1—C2—H2	−179.4
C7—C1—C2—C3	−179.53 (16)	C7—C1—C2—H2	0.5
C1—C2—C3—C4	1.0 (3)	C1—C2—C3—H3	−179.0
C2—C3—C4—C5	−1.1 (3)	H2—C2—C3—H3	1.0
C3—C4—C5—C6	−0.6 (3)	H2—C2—C3—C4	−179.0
C2—C1—C6—N1	178.70 (14)	C2—C3—C4—H4	178.9
C7—C1—C6—N1	−1.2 (2)	H3—C3—C4—H4	−1.1
C2—C1—C6—C5	−2.2 (2)	H3—C3—C4—C5	178.9
C7—C1—C6—C5	177.95 (14)	C3—C4—C5—H5	179.4
C4—C5—C6—N1	−178.71 (16)	H4—C4—C5—H5	−0.6
C4—C5—C6—C1	2.2 (3)	H4—C4—C5—C6	179.4
C6—C1—C7—O1	−178.98 (15)	H5—C5—C6—C1	−177.8
C2—C1—C7—O1	1.1 (2)	H5—C5—C6—N1	1.3
C6—C1—C7—C8	1.0 (2)	C7—C8—C9—H9	178.8
C2—C1—C7—C8	−178.88 (14)	C12—C8—C9—H9	1.4
O1—C7—C8—C9	−179.87 (16)	H9—C9—N1—C10	−2.9
C1—C7—C8—C9	0.1 (2)	H9—C9—N1—C6	−178.9
O1—C7—C8—C12	−2.7 (3)	H10A—C10—C11—H11A	−54.6
C1—C7—C8—C12	177.27 (14)	H10A—C10—C11—H11B	−174.6
C7—C8—C9—N1	−1.2 (3)	H10A—C10—C11—H11C	65.4
C12—C8—C9—N1	−178.55 (15)	H10B—C10—C11—H11A	63.1
C9—C8—C12—O2	−1.1 (3)	H10B—C10—C11—H11B	−56.9
C7—C8—C12—O2	−178.36 (17)	H10B—C10—C11—H11C	−176.9
C9—C8—C12—N3	177.80 (15)	N1—C10—C11—H11A	−175.7
C7—C8—C12—N3	0.6 (2)	N1—C10—C11—H11B	64.3
N2—C13—C14—C15	−176.91 (16)	N1—C10—C11—H11C	−55.7
N2—C13—C14—C18	1.9 (3)	H10A—C10—N1—C6	−43.9
C18—C14—C15—C16	−0.8 (3)	H10A—C10—N1—C9	140.2
C13—C14—C15—C16	178.15 (16)	H10B—C10—N1—C6	−161.6
C14—C15—C16—N4	0.2 (3)	H10B—C10—N1—C9	22.5
N4—C17—C18—C14	−0.6 (3)	C8—C12—N3—H3N	1.0 (13)
C15—C14—C18—C17	0.9 (3)	H13—C13—C14—C15	3.1
C13—C14—C18—C17	−177.93 (17)	H13—C13—C14—C18	−178.1
C8—C9—N1—C6	1.1 (2)	H13—C13—N2—N3	−1.5
C8—C9—N1—C10	177.08 (16)	C13—C14—C15—H15	−1.8
C1—C6—N1—C9	0.2 (2)	C18—C14—C15—H15	179.2
C5—C6—N1—C9	−178.95 (15)	C13—C14—C18—H18	2.1
C1—C6—N1—C10	−175.74 (15)	C15—C14—C18—H18	−179.1
C5—C6—N1—C10	5.1 (2)	C14—C15—C16—H16	−179.8
C11—C10—N1—C9	−98.68 (19)	H15—C15—C16—H16	0.2
C11—C10—N1—C6	77.3 (2)	H15—C15—C16—N4	−179.8
C14—C13—N2—N3	178.47 (14)	H16—C16—N4—C17	−179.7
O2—C12—N3—N2	−0.3 (3)	H17—C17—C18—C14	179.4
C8—C12—N3—N2	−179.21 (14)	H17—C17—C18—H18	−0.6
C13—N2—N3—C12	−178.62 (16)	N4—C17—C18—H18	179.4
C15—C16—N4—C17	0.3 (3)	H17—C17—N4—C16	180.0
C18—C17—N4—C16	0.0 (3)	C13—N2—N3—H3N	1.2 (14)
C6—C1—C2—H2	−179.43 (1)	O2—C12—N3—H3N	179.9 (13)
C7—C1—C2—H2	0.46 (2)		

### Hydrogen-bond geometry (Å, °)

**Table:**

*D*—H···*A*	*D*—H	H···*A*	*D*···*A*	*D*—H···*A*
N3—H3N···O1	0.92 (2)	1.85 (2)	2.639 (2)	143.1 (17)
C10—H10A···O2^i^	0.97	2.46	3.398 (2)	163

Symmetry codes: (i) *x*+1/2, −*y*+1/2, *z*−1/2.
